# Conservative Fragments in Bacterial 16S rRNA Genes and Primer Design for 16S Ribosomal DNA Amplicons in Metagenomic Studies

**DOI:** 10.1371/journal.pone.0007401

**Published:** 2009-10-09

**Authors:** Yong Wang, Pei-Yuan Qian

**Affiliations:** 1 KAUST Global Partnership Program, Department of Biology, Hong Kong University of Science and Technology, Clear Water Bay, Hong Kong, China; 2 King Abdullah University of Science and Technology, Jeddah, Saudi Arabia; NERC Centre for Ecology and Hydrology, United Kingdom

## Abstract

Bacterial 16S ribosomal DNA (rDNA) amplicons have been widely used in the classification of uncultured bacteria inhabiting environmental niches. Primers targeting conservative regions of the rDNAs are used to generate amplicons of variant regions that are informative in taxonomic assignment. One problem is that the percentage coverage and application scope of the primers used in previous studies are largely unknown. In this study, conservative fragments of available rDNA sequences were first mined and then used to search for candidate primers within the fragments by measuring the coverage rate defined as the percentage of bacterial sequences containing the target. Thirty predicted primers with a high coverage rate (>90%) were identified, which were basically located in the same conservative regions as known primers in previous reports, whereas 30% of the known primers were associated with a coverage rate of <90%. The application scope of the primers was also examined by calculating the percentages of failed detections in bacterial phyla. Primers A519–539, E969–983, E1063–1081, U515 and E517, are highly recommended because of their high coverage in almost all phyla. As expected, the three predominant phyla, Firmicutes, Gemmatimonadetes and Proteobacteria, are best covered by the predicted primers. The primers recommended in this report shall facilitate a comprehensive and reliable survey of bacterial diversity in metagenomic studies.

## Introduction

In prokaryotes, the 16S ribosomal RNA (rRNA) genes are essential and occur in at least one copy in a genome [1]. They are also present in all mitochondrial genomes, which have lost most of their ancestral gene content in the long evolutionary history of symbiosis [2]. The universality of the genes makes them an ideal target for phylogenetic studies and taxonomic classification [3]. The products of the rRNA genes can fold into a complex, stable secondary structure, consisting of stems and loops [4]. The sequences of some of the loops are conservative across nearly all bacterial species because of the essential functions involved, whereas the features of the structural parts are largely variant and specific to one or more classes [5], [6]. Since the invention of the polymerase chain reaction (PCR) technique [7], the variant regions, V1–V9, of the 16S rRNA genes (rDNAs) have been used for species identification [8].

The appropriate primers for a PCR reaction are critical because an over-relaxed match between a primer and its target leads to PCR failure. For 16S rDNAs, the primers (15–20 nucleotides (nt)) are located in the conservative regions that flank a target region used for phylogenetic analysis [8]. The first sets of primers were designed by using conservative regions of 16S rDNA sequences from different species and were named according to their positions on *Escherichia coli* 16S rDNA [8]; this has become the protocol for subsequent primer design. For example, primer E685 corresponds to eubacterial P4 region [9] and primer A344 targets the archaeal H339 region [10]. In the recent decades, more primers have been designed for bacterial studies with tools such as ARB [11], as the number of known 16S rDNA sequences increases. Moreover, primers targeting a specified phylum have recently been designed [12]. However, known polymorphisms also accumulate in the conservative regions, when a large number of 16S rDNA sequences were generated and deposited in public databases, such as the Ribosomal Database Project (RDP) database [13]. Consequently, the originally widely used primers may not be suitable for a small group of bacteria, as noticed in recent studies [14], [15], [16].

The problem of primer selection is even more difficult and has attracted attention because of recent advances in metagenomic studies. Massive parallel sequencing techniques allow unprecedentedly rapid and economical DNA sequencing. Nearly one million sequences of 400 nt can be generated by the Roche 454 FLX Titanium machine, allowing the deep sequencing of environmental bacterial genomes [17]. In many experiments, amplicons of the V3 and/or V6 regions have been subjected to the pyrosequencing [18]. These two variant regions in 16S rDNA can provide sufficient phylogenetic information about the bacteria in samples [19], [20].

The accumulation of known polymorphisms in the conserved regions means that the coverage rates of some primers are declining [6]. This might cause problems in using widely accepted primers if they fail to recover a high percentage of bacterial species in uncultured environmental samples, as expected. Using wrong primers will lead to failure to detect some bacterial species and consequently incomplete surveys in metagenomic studies.

Previous studies have found that Archaea- and Eubacteria-specific primers cannot target a spectrum of classes [14], [16]. The known primers for the Archaea are not always suitable for amplifying the 16S rRNA amplicons for Korarchaeota or Nanoarchaeota [16]. Using the RDP classifier and the BLAST program, Baker et al. (2003) and Huws et al. (2007) have investigated the species specificity and coverage spectrum of the known primers. However, the results of both studies are preliminary in that the coverage rates of the primers were not given. Moreover, the latter study did not consider degeneracies in these primers. In a recent work, the coverage of several known primers was surveyed using several sets of metagenomic data, and the primers with better performance were recommended for future work [21]. All these studies used known primers and provided brief information of their phylum specificity. But we still do not have a ranking of the capacities of the known primers useful for environmental samples and a list of all candidate primers for bacterial 16S rRNA genes.

In this study, we identified conservative fragments in 16S rRNA genes from the RDP database and compiled a list of candidate primers. The predicted primers reported in this study comprise nearly a full-set of primers for prokaryotic 16S rRNA genes and largely overlapped with known primers, regardless of any shift in positions. The average coverage rate of our primers is 96%, markedly higher than that of other known primers. We also studied the scope of their application, which should provide guidance for metagenomic studies.

## Results

### Designing predicted primers using conserved fragments of 16S rDNA sequences

We identified continuous conservative sites (>14 nt) in the Archaea and Eubacteria separately. They were positioned on the *E. coli* 16S rRNA gene by using a pairwise alignment and converted to conservative fragments. There were 8 archaea-specific and 11 eubacteria-specific conservative fragments of various lengths. Most of the conservative archaeal and eubacterial fragments were numbered according to approximate positions on the *E. coli* 16S rRNA gene, and only four fragments lacked any counterparts: eubacterial fragments 104–120, 683–707, and 1177–1197, and archaeal fragment 1225–1242 (Table 1). Among the overlapping fragments, we found obvious sequence variations such as between archaeal 344–367 and eubacterial 314–368. The differences in these fragments possibly reflect the major characteristics of the functional parts of the 16S rRNA transcripts, which probably developed after the divergence of the Archaea and Eubacteria.

**Table 1:** The conservative fragments in archaeal and eubacterial 16S rDNAs.

Bacteria	Position	Conservative fragment
E	104–120	GGCGVACGGGTGAGTAA
E	314–368	CAYTGGRACTGAGACACGGYCCARACTCCTACGGG
		AGGCAGCAGTRRGGAATHTT
A	344–367	AYGGGGYGCAGCAGGCGRGAAARC
E	505–539	GGCTAACTHCGTGCCAGCAGCCGCGGTAATACGDA
A	506–547	GGYAAGDCYGGTGYCAGCCGCCGCGGTAAHACCRC
		CDRTGGCGAA
E	683–707	GTGTAGRGGTGAAATKCGYAGAKAT
E	764–806	CGAAAGYGTGGGKAKCRCAGGATTAGATACCCTGGT
		AGTCC
A	779–806	CRAACSGGATTAGATACCCSGGTAGTCC
E	879–893	CCRCCTGGGGAGTAC
A	882–936	CCTGGGRAGTACGKHCGCAAGDRTGAAACTTAAAGG
		AATTGGCGGGGGAGCAC
E	909–940	ACTCAAAKGAATTGACGGGGRCCCGCACAAGC
A	947–973	GCSTGCGGYTYAATTGGABTCAACGCC
E	949–964	ATGTGGTTTAATTCGA
E	969–985	ACGCGARGAACCTTACC
A	1043–1073	GAGAGGWGGTGCATGGCCGYCGYCAGYTCGT
E	1048–1114	GTGSTGCATGGYTGTCGTCAGCTCGTGYCGTGAGRT
		GTYGGGTTAAGTCCCRYAACGAGCGCAACCC
A	1094–1111	GTCAGRYAACGARCGAGA
E	1177–1197	GGAAGGYGGGGAYGACGTCAA
A	1225–1242	ACACGCGSGCTRCAAWGG

The conservative fragments were generated from multiple alignments among 6,624 Archaea (A) and 275,057 Eubacteria (E) in RDP database. The positions are determined according to relative positions in *E. coli* 16S rDNA genome. Y: C or T; R: A or G; W: A or T; K: G or T; M: C or A; S: C or G; V: not T; H: not G; B: not A; D: not C.

**Figure 1:**
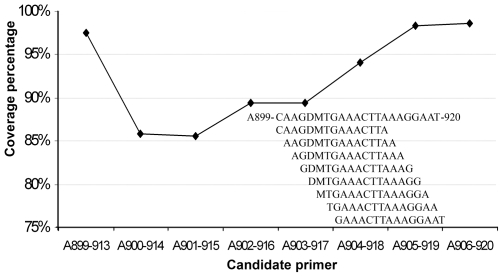
Coverage rates of candidate primers within a conservative fragment. The coverage rates (%) of eight candidate primers within the conservative fragment 5′-CAAGDMTGAAACTTAAAGGAAT-3′ were determined using all archaeal 16S rDNA sequences (>1,200 nt) as the reference dataset. The coverage rate is the percentage of the rDNA sequences that have a target fragment matching a given candidate primer. One mismatch is allowed in the match.

Next, we selected candidate primers (15 nt) from the fragments by checking their coverage rates. A high coverage rate indicates a high percentage of bacteria in our dataset with a target site for the candidate primer. Every candidate primer was examined with a sliding window, which was moved across the fragments (Fig. 1). Although all the sites were highly conservative, the coverage rates of the candidate primers on the same fragment varied markedly and might be distributed across a larger range than that shown in Figure 1. The candidate primers containing degenerate sites clearly corresponded to low coverage rates (Fig. 1), suggesting that introduction of the degeneracies could not ensure complete matches between the primers and their targets, and that the degeneracies by themselves pointed to the positions of weak sites in the candidate primers as well as in the conservative fragments.

After we filtered out the candidate primers with a coverage rates below 90%, the remaining overlapping primers were merged again and new coverage rates were measured for them (Table 2). Thirty candidate primers (13 for the Archaea and 17 for the Eubacteria) were identified and are of potential use in designing forward and reverse primers. Notably, eubacterial conservative fragment 104–120 did not contain candidate primers that met the selection criteria. Some primers for the Archaea and Eubacteria were not only numbered with the same *E. coli* rDNA positions but were also highly homologous in their pattern. Therefore, they were defined as predicted universal primers: U515–532, U785–800, U909–928, and U1052–1071 (Table 2).

**Table 2:** The coverage rate of predicted primers.

Bacteria	Position	Sequence	Average rate	Coverage rate
E	321–336	ACTGAGACACGGYCCA	95.7%	96.1%
E	329–343	ACGGYCCARACTCCT	95.3%	96.0%
E	338–358	ACTCCTACGGGAGGCAGCAGT	97.3%	96.3%
A	346–361	GGGGYGCAGCAGGCG	94.2%	94.3%
E	350–364	GGCAGCAGTRRGGAA	95.1%	95.5%
E	505–524	GGCTAACTHC GTGCCAGCAG	95.3%	95.1%
A	514–528	GGTGYCAGCCGCCGC	97.3%	98.5%
E	515–532	GTGCCAGCAGCCGCGGTA	92.6%	91.0%
U	515–532	GTGYCAGCMGCCGCGGTA	-	96.9%/96.9%
A	519–539	CAGCCGCCGCGGTAAHACCRC	96.7%	97.1%
E	683–700	GTGTAGMGGTGAAATKCG	92.6%	90.5%
E	783–797	CAGGATTAGATACCC	97.9%	97.9%
E	785–806	GGATTAGATACCCTGGTAGTCC	95.9%	94.6%
A	785–800	GGATTAGATACCCSGG	98.1%	98.4%
U	785–800	GGATTAGATACCCBGG	-	98.4%/97.1%
A	884–898	TGGGRAGTACGKHCG	97.1%	97.1%
A	899–913	CAAGDMTGAAACTTA	97.6%	97.6%
A	905–920	TGAAACTTAAAGGAA	98.3%	98.3%
A	921–936	TTGGCGGGGGAGCAC	98%	97%
E	909–926	ACTCAAAKGAATTGACGG	98.5%	97.9%
U	909–928	ACTYAAAKGAATTGRCGGGG	-	93.2%/92.1%
E	919–939	ATTGACGGGGRCCCGCACAAG	96.3%	96.1%
A	947–964	GCSTGCGGYTYAATTGGA	91.6%	90.5%
E	949–964	ATGTGGTTTAATTCGA	93.5%	93.5%
A	958–973	AATTGGABTCAACGCC	90.6%	93.5%
E	969–984	ACGCGARGAACCTTAC	97.4%	97.1%
A	1045–1059	GAGGWGGTGCATGGC	95.7%	97.4%
A	1052–1071	TGCATGGCCGYCGYCAGYTC	96.6%	95.1%
E	1052–1072	TGCATGGYTGTCGTCAGCTCG	97.1%	99.0%
U	1052–1071	TGCATGGYYGYCGYCAGYTC	-	95.1%/98.8%
E	1063–1081	CGTCAGCTCGTGYCGTGAG	99.2%	99.3%
E	1096–1114	CCCRYAACGAGCGCAACCC	96.8%	95.6%
E	1177–1193	GGAAGGYGGGGAYGACG	98.2%	98.2%
A	1226–1242	CACGCGSGCTRCAAWGG	93.8%	93.5%

The primers in this table were fragments within the conservative fragments in Table 1. If coverage rates of neighboring candidate primers were all above 95%, they were merged. If no predicted primers in a fragment, the cutoff rate decreased to 90%. The average coverage rate was thus calculated for the neighboring primers. Universal primers (U) were obtained by referring to archaeal (A) and eubacterial (E) predicted primers at the same positions of *E. coli* genome. The coverage rate was measured for the merged primer. For the universal primers, both were provided (A/E). Abbreviated names for bacteria and the positions were listed as those in Table 1.

### Coverage rates of predicted and known primers

To evaluate the accuracy of our prediction, the predicted primers were compared with 29 known primers including 13 Archaea-specific, 9 Eubacteria-specific, and 7 universal primers (Table 3). After cleaning the overlapping primers, we found that our predicted primers contained a novel primer, A884–898, which has not been reported previously. Although nearly all the predicted and known primers were located in the same regions, some of the known primers were probably problematic because of the lack of sufficient degeneracies and the low degree of conservation at some sites in the primers. Therefore, the coverage rates of these primers were compared with those of the predicted primers.

**Table 3:** Coverage rate of known primers.

Primer [Ref]	Primer sequence 5′-3′	Position	Coverage rate
A333F [16]	TCCAGGCCCTACGGG	333–348	57.4%
E334F [14]	CCAGACTCCTACGGGAGGCAGC	334–356	74.2%
A340F [16]	CCCTACGGGGYGCASCAG	340–358	88.3%
U341F [16]	CCTACGGGRSGCAGCAG	341–358	91.1%/96.9%
E343F [20]	TACGGRAGGCAGCAG	343–357	98.7%
A344F (A) [15]	GGGGYGCASCAGGSG	344–360	90.8%
A344F (B) [15]	ACGGGGCGCAGCAGGCGCGA	344–363	74.2%
U515F [16]	GTGCCAGCMGCCGCGGTAA	515–534	63.3%/99.0%
E517F [20]	GCCAGCAGCCGCGGTAA	517–533	99.1%
A519R [15]	GGTDTTACCGCGGCKGCTG	519–537	98.0%
A519F [15]	CAGCMGCCGCGGTAA	519–533	98.6%
U519F [16]	CAGCMGCCGCGGTAATWC	519–537	96.7%/98.5%
A685R [27]	TTACGGGATTTCACTCCTAC	685–704	19.5%
E685R [28]	ATCTACGCATTTCACCGCTAC	685–705	79.8%
U779F [16]	GCTAASSGGATTAGATACCC	779–799	89.9%/5.0%
E786F [14]	GATTAGATACCCTGGTAG	786–803	95.2%
U789F [16]	TAGATACCCSSGTAGTCC	789–807	97.7%/94.8%
A806R [15]	GGACTACVSGGGTATCTAAT	787–806	96.4%
E806R [14]	GGACTACCAGGGTATCTAAT	787–806	95.1%
U906F [16]	GAAACTTAAAKGAATTG	906–923	98.3%/54.2%
A906R [15]	CCCGCCAATTCCTTTAAGTTTC	906–927	97.3%
E917F [20]	GAATTGACGGGRCCC	917–932	92.5%
A915R [15]	GTGCTCCCCCGCCAATTCCT	915–934	97.1%
E939R [14]	CTTGTGCGGGCCCCCGTCAATTC	917–939	93.1%
A976R [16]	CCGGCGTTGAMTCCAATT	957–976	92.7%
A1040F [16]	GAGAGGWGGTGCATGGCC	1040–1058	95.2%
U1053F [16]	GCATGGCYGYCGTCAG	1053–1068	97.2%/97.2%
A1098F [16]	GGCAACGAGCGMGACCC	1098–1115	67.0%
E1099F [20]	GYAACGAGCGCAACCC	1099–1114	97.0%

The source of the known primers is labeled. The degenerated sites are defined in Table 1. The names of Archaea-specific, Eubacteria-specific, and universal primers are started with ‘A’, ‘E’ and ‘U’, respectively. For the universal primers, the coverage rates for both the Archaea and Eubacteria are given (A/E).

For the predicted primers, the average coverage rates of the archaeal and eubacterial primers were 96% and 96.2%, respectively. The average coverage rate of the predicted universal primers was 96%. The values for the known archaeal, eubacterial, and universal primers were 85%, 77.4%, and 84.3%, respectively. Overall, the coverage rates of all the predicted primers were above 90%, whereas the coverage rates of the 11 known primers (30.6% of all known primers) were lower than 90% (Table 3). The coverage rates of the predicted primers were significantly higher than those of the known primers (Spearman test; *P*<0.00001). Our results also cast doubt on the validity of some known universal primers, as three out of the seven showed poor coverage in Archaea or Eubacteria: the coverage rate of U779 in Archaea was only 5%. The remaining primers, U341F, U519F, U789F, and U1053F, are highly recommended for their high coverage rates in all bacteria. U341F was not included among our predicted universal primers, as polymorphisms and dissimilarities in this region would introduce too many degeneracies when both the Archaea and Eubacteria are considered.

### Phylum specificity of predicted and known primers

As described above, we generated a list of predicted and known primers with a high coverage rate for both the Archaea and Eubacteria. However, it was a challenge to amplify the 16S rRNA sequences of all the bacteria in environmental samples. Generally, the dominant and well-characterized bacterial phyla could be detected easily according to the principles of primer design. The problem was how to identify the minority bacterial phyla; occasionally, a whole phylum was missed. In the RDP database, the numbers of bacteria from different phyla differed substantially, and the failure to detect a small phylum might simply result in less than 1% loss of coverage rate. Therefore, it was necessary to assess the phylum specificity of our predicted primers, as a supplementary evaluation other than coverage rate.

**Figure 2:**
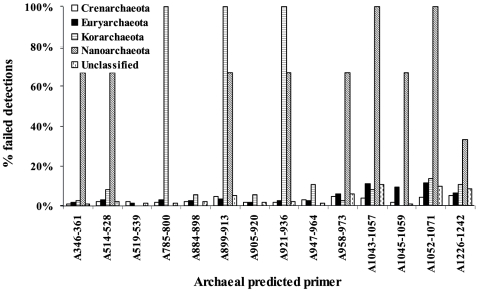
Phylum specificity of predicted primers for Archaea. Information on the primers is listed in Table 2. The primers were used to find their targets in 1544 Crenarchaeota, 4153 Euryarchaeota, 37 Korarchaeota, 3 Nanoarchaeota, and 878 unclassified species. The sequences without a target were classified into different phyla.

We first displayed coverage spectrum of 13 Archaea-specific primers. In the Crenarchaeota and Euryarchaeota, the percentage of failed detections was below 10% for the primers, indicating that the coverage of these Archaea was rather stable (Fig. 2). However, the coverage of Korarchaeota and Nanoarchaeota varied remarkably in a range of 0%–100%. Primers A785–800, A899–913, and A905–936 were not suitable for Korarchaeota, as indicated by their 100% of failure rates. The highly variant coverage rates of these primers in Nanoarchaeota were not surprising because there were only three representatives of this taxon (>1200 nt) in the database. In light of the spectrum found in this test, A519–539 could provide the best coverage of all archaeal phyla. Although some primers failed to cover Korarchaeota completely, they provided location information for the design of Korarchaeota-specific primers. Among the 12 known Archaea-specific and universal primers examined, U906F and U1053F performed better than the others (Fig. S1). And the result confirms that the Archaea-specific primers do not have high coverage rates in Korarchaeota and/or Nanoarchaeota.

**Figure 3:**
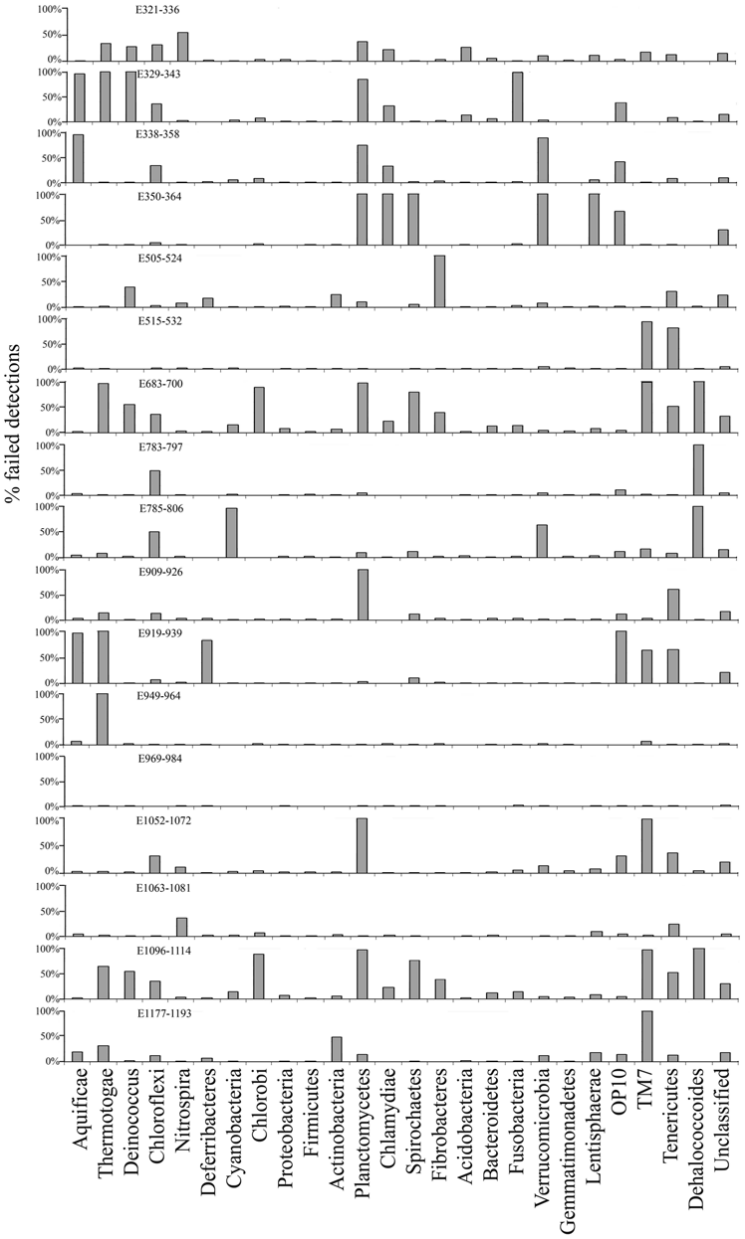
Phylum specificity of predicted primers for Eubacteria. Information on the primers is listed in Table 2. The primers were used to find their targets in 778 Aquificae, 325 Thermotogae, 550 Deinococcus, 1922 Chloroflexi, 651 Nitrospira, 245 Deferribacteres, 4655 Cyanobacteria, 195 Chlorobi, 91629 Proteobacteria, 94475 Firmicutes, 23266 Actinobacteria, 2274 Planctomycetes, 216 Chlamydiae, 2646 Spirochaetes, 202 Fibrobacteres, 4040 Acidobacteria, 33924 Bacteroidetes, 737 Fusobacteria, 2851 Verrucomicrobia, 441 Gemmatimonadetes, 108 Lentisphaerae, 114 OP10, 327 TM7, 1683 Tenericutes, 108 Dehalococcoides, and 6333 unclassified bacteria. The phyla with less than 100 sequences were ignored. The sequences without a target were classified into different phyla.

The same test was performed with 17 predicted Eubacteria-specific primers on 25 eubacterial phyla. Most of the primers showed a weakness in finding targets in a small spectrum of eubacteria phyla (Fig. 3). E969–983 was the best primers because it displayed the lowest average rate (1%) of failed detections, followed by E1063–1081 with an average failure percentage of 4.6%. The highest average failure percentage (32.8%) was observed for E1177–1193. Surprisingly, the difference between E783–797 and E785–806 was 9%, although the major part of E783–797 lies within E785–806 except for the first two nucleotides. Therefore, different primers show clear phylum specificity, and fine adjustment of the primer target could achieve better coverage. This was verified by variant rates of failed detections observed for the same phylum dataset using different primers. We thus measured the average of the rates for individual phyla to determine the bacteria phyla that were most easily detected, and the results showed that Firmicutes, Gemmatimonadetes and Proteobacteria were the phyla with the highest rates of match to the primers. In ascending order, the average percentages of failed detections were 1.47%, 1.54%, and 1.9%, respectively, for three phyla. In contrast, Planctomycetes and TM7 were associated with the highest average rates of failed detections (40% and 31.8%, respectively) with large standard deviation (42% and 43%, respectively), indicating that the coverage of the primers in these two phyla is not stable. These results could be foreseen because the overwhelming number of representatives from Firmicutes and Proteobacteria (Fig. 3) caused a bias in primer design. The polymorphisms in the minority phyla were largely ignored, leading to insufficient degeneracies in the primers.

The performance of known primers was also assessed. Of the top three phyla, Firmicutes and Proteobacteria were most easily detected with the known primers (Fig. S2). A minor phylum, Deferribacteres, was the phylum best covered by the known primers, with the lowest average rate (0.45%) of failed detections, followed by Deinococcus and Acidobacteria. This finding suggests that the 16S rDNA sequences collected previously from the RDP and GenBank were less biased in collection of certain phyla. However, the usefulness of the known primers for Verrucomicrobia was limited, and half the known primers showed >50% failed detections, perhaps reflecting the lack of representatives of this phylum when the primers were designed. Among the known primers, U515 and E517 are highly recommended in light of their wide spectrum of perfect coverage. E1099F also had an overall high coverage rate, although it failed to detect most of Planctomycetes (Fig. S2).

### Assessment of Cyanobacteria-specific primers

The above results are useful for studies that focus on a specific phylum. By designing primers for a phylum of interest, only the 16S rDNA of the desired bacterial species is amplified for subsequent studies. We examined three Cyanobacteria-specific primers, CYA106F, CYA359F, and CYA781R [12]. The coverage rate for all Eubacteria was 31.7% for primer CYA106F, 7.4% for CYA359F, and 2.3% for CYA781R. We classified the identified bacteria species and found that CYA106F was not specific for the Cyanobacteria. CYA106F, CYA359F and CYA781R could be used to identify 80%, 98%, and 92% of the 4655 Cyanobacterial sequences in our collection, independently. Moreover, CYA106F and CYA359F had many targets in Firmicutes: 75% of 94475 Firmicutes sequences were targets of CYA106F and 9% were targets of CYA359F. However, CYA781R had an extremely low coverage rate (0.001%) in Firmicutes. An appropriate combination of forward and reverse primers could avoid generating a mixture of amplicons from Firmicutes. These primers designed, based on previous database collection, are still useful today.

### Distance of the primers to variant regions of 16S rRNA genes

**Figure 4:**
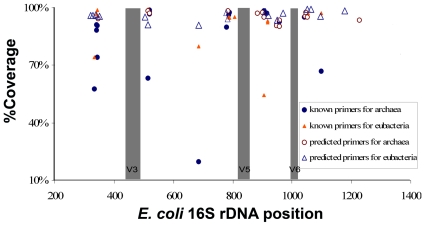
Primer distributions and distances to variant regions. The V3, V5, and V6 regions are variant regions of the 16S rRNA genes. Their locations on the *E. coli* 16S rRNA gene were shown as shadowed regions. The relative distances of the primers (including the known and predicted primers) to the three regions were shown.

We put the predicted and known primers onto the same map to compare their relative distances to the 16S rRNA variant regions. Three of these regions (V3, V5, and V6) in *E. coli* are shown in Figure 4. The primers were concentrated in six narrow regions, spanning the three variant regions. For those primers with high coverage rates, the predicted and known primers overlapped strongly. The “hot” regions where the primers bind were: 321–364, 505–539, 783–806, 884–939, 947–984, and 1045–1081. The sizes of the amplicons from the V3 region and V5–V6 region were about 180 nt and 270 nt, respectively. Both could be completely sequenced with the 454 FLX platform.

## Discussion

In this study, we predicted all the potential primers for bacterial 16S rDNA amplicon. Their positions are largely consistent with those of known primers, but the average coverage rate is higher than that of known primers. Some of the known primers used in previous studies have been found to be unsuitable for the amplification of 16S rDNA fragments from uncultured samples. We also confirmed that most of the primers in hand are highly specific for a spectrum of bacterial species, and definitely cannot be used to amplify all bacteria in uncultured samples. Our result should be helpful in the design of primer for species-specific amplicons, when research interests are restricted to a certain species. As well as from 16S rDNAs, the protocol provided in this study can also be applied to the detection of genetic variations in other essential genes in bacterial communities [22], all of which are important in metagenomic studies.

With recent advances in massively parallel sequencing techniques, the bacteria world in untouched ecological niches can be explored to survey its biodiversity and niche-specific metabolic pathways. The use of 16S rDNA amplicon sequencing allows us to estimate the abundance and diversity of these bacteria, whereas the exhaustive detection of rare species is difficult to achieve. In recent metagenomics studies, the number of phylotypes in the same number of 16S rDNA sequences varied substantially for samples from different environments and geographical sites [23], [24], [25]. Despite this, we cannot exclude the possibility that amplification efficacy of the different 16S rDNA primers used in these studies led to the underestimation of bacterial richness. Primer usage is undoubtedly one of the most critical limiting factors affecting 16S rDNA analysis [18]. Although V3 and V6 are the most popular regions examined in recent metagenomic studies, the primers used differ [18]. This may lead to different capture depths of the bacteria in environmental samples, attributable to varying amplification efficiencies and coverage rates of the primers.

In an attempt to compare the results of different studies, research groups have tended to use the same primers. In several studies of microbial communities in the human gut and seawater, primers 967F and 1046R have been used to amplify the V6 region to avoid the bias caused by primer selection [19], [23], [25], [26]. Our study provides a reliable set of candidate primers for researchers to achieve an approximately full coverage of bacterial 16S rDNAs and comparable results among different studies.

The recently updated Roche 454 Titanium platform yields about one million reads per run, with reads up to about 400 nt [17]. The increase in read lengths allows us to analyze longer amplicons from the variant regions of 16S rDNAs. However, we are still far from being able to sequence amplicons spanning both V3 and V6 (Figure 4). Among all the primers discussed, E683–700 is important because it can be used as a reverse primer to generate amplicons of ∼340 nt from the V3 region or a forward primer designed to generate ∼400 nt amplicons spanning the V5 and V6 regions. The closest primers to it are at least 100 nt away, and it is the only primer that allows the full utilization of the sequencing capacity of the new 454 platform. However, a potential problem is its relatively low coverage rate of 91% for the Eubacteria. Notably, no predicted or known primer has been found for the Archaea in this region. Therefore, the amplicons obtained with E683–700 from an uncultured environmental sample will specifically belong to Eubacteria.

One limitation of this study is that the primer design depended on the data in the RDP database. The bacteria in rare biospheres can never be identified if the employed primers are not applicable to them. New primers cannot be invented in case of lack of representatives of those bacteria in the RDP database. Although numerous 16S rRNA genes had been collected in databases, the real bacterial world in environmental samples will still be invisible under the current protocol for 16S rDNA detection. In this study, three nearly full-length Nanoarchaeota were used as references for primer design. The unstable coverage rate observed is an obstacle to evaluating the efficiency of the predicted and known primers at all sub-levels. Fortunately, ongoing and completed metagenomic projects may help us by providing nearly full-length 16S rRNA genes and by increasing the representatives of the rRNA genes particularly from rare biospheres. As the number of 16S rRNA genes increases, we can gain unprecedented knowledge about the formation and dynamics of bacterial communities in different ecological niches.

Another limitation of this study lies in dependence on the data collection of the RDP database. The accuracy of our prediction of the phylum specificity of the primers was affected by the numbers of orders and classes in a given phylum and the sequences available for these sub-phylum levels. If a phylum has one class with sequences concentrated in one order, the coverage rate of the primers will probably be higher than for a phylum with many classes, all having a few sequences in different orders.

## Materials and Methods

We downloaded nearly full-length 16S rRNA gene sequences (≥1200 nt) from the website of the RDP (version 10.8) [13]. The dataset included 6,624 archaeal and 275,057 eubacterial sequences in the alignment model.

For a sequence in the aligned format, we defined the sites as one of three types: conservative sites, polymorphic sites, and gaps. A conservative site consisted of one or two highly predominant nucleotides, but occasionally three nucleotides at nearly equal frequencies. A conservative site with two or three predominant nucleotides was called a “polymorphic conservative site”. A small proportion of sequences did not have a nucleotide for alignment, and a gap was inserted at that site. A polymorphic site contains variant nucleotides in a small proportion of sequences, and a gap was inserted at the corresponding position in the remaining sequences. The conservative and polymorphic sites differ in the proportion of the gaps. We obtained conservative fragments (>14 nt) of 16S rDNA for the Archaea and Eubacteria separately, by removing the polymorphic sites and retaining the conservative sites. A polymorphic site was removed if the proportion of the sequences with a gap was higher than 95%; a conservative site was recorded if the proportion was below 10%. We used a sliding window of 30 nt, moving across the aligned sequences in steps of 10 nt. If there were more than 14 continuous conservative sites in a window, a conservative fragment was detected. We also limited the maximum numbers of polymorphic conservative sites in each sliding window to three. In the subsequent manual adjustments, the conservative fragments were merged if they were overlapped. Because of the high divergence of the rDNAs of the Archaea and Eubacteria [16], the search for conservative fragments using all the rDNAs could not detect those specific to the Eubacteria or Archaea. We finally obtained two sets of conservative fragments, one each for the Archaea and Eubacteria.

We then calculated the coverage rates of the conservative fragments to obtain candidate primers with which to generate 16S rDNA amplicons. The primary requirements for a candidate primer were a length of 15 nt and no polymorphisms within the first four or last four sites. We listed all the 15-nt candidate primers in a conservative fragment and then determined their coverage rates using the RDP bacterial sequences. Before this was done, the alignment model of the sequences was transformed to the original FASTA format by removing the gaps. We detected the matching part of each bacterial sequence by using the first four or last four nucleotides (if the first four nucleotides had polymorphisms, the last four were used) as a seed. Once the seed met a matching one, we extended the matching part back and forth until the full-length candidate primer was matched to a certain part of the sequence. For polymorphisms, all nucleotides were tested to see if they matched the nucleotide at the target site. We allowed at most one mismatch between the candidate primer and its target. Finally, the coverage rate of the candidate primer was measured as a percentage of the sequences in which the target was found.

With respect to the coverage rate of the primers, we merged the neighboring candidate primers while maintaining the average rate above 95%. When a conservative fragment did not contain any eligible candidates, the threshold coverage rate decreased to 90%. Universal primers are those sequences common to the archaeal and eubacterial predicted primers. After merging, the predicted primers were compared with known primers from previous reports [14], [15], [20], [27]. The known primers positioned at sites numbering >1200 nt on the *E. coli* 16S rDNA were not included, because some of the sequences in our collection were not within the coverage scope of the primers. The RDP bacterial sequences were used as a reference dataset to determine the coverage rates of the known primers. For the predicted and known primers, we pooled bacterial species in which the primers did not have their targets. The bacteria were then sorted with SEQCART in the RDP database, and the percentage of failed detections was calculated for each bacterial phylum.

Finally, three Cyanobacteria-specific primers, CYA106F, CYA359F, and CYA781R [12] were evaluated. These primers were designed based on cyanobacterial sequences from the RDP and GenBank, and were examined with a larger dataset from the current version of RDP database (10.8).

## Supporting Information

Figure S1 Phyla specification of known primers for archaea. Legend: The known primers were used to find their targets in a RDP dataset consisting of 1544 Crenarchaeota, 4153 Euryarchaeota, 37 Korarchaeota, 3 Nanoarchaeota and 878 unclassified species. The sequences without a target were classified into different phyla. The sequences and sources of the known primers are referred to Table 3.
(3.16 MB TIF)

Figure S2 Phyla specification of known primers for eubacteria. Legend: The sequences and sources of the known primers are shown in Table 3. The details of the RDP dataset in which the primers were used to find their targets are referred to Figure 3.
(4.68 MB TIF)
