# Supplementary figures and images for: Conservative Fragments in Bacterial 16S rRNA Genes and Primer Design for 16S Ribosomal DNA Amplicons in Metagenomic Studies
Source: PLoS One. 2009 Oct 9;4(10):e7401. doi: 10.1371/journal.pone.0007401 (PMC2754607; doi:10.1371/journal.pone.0007401)

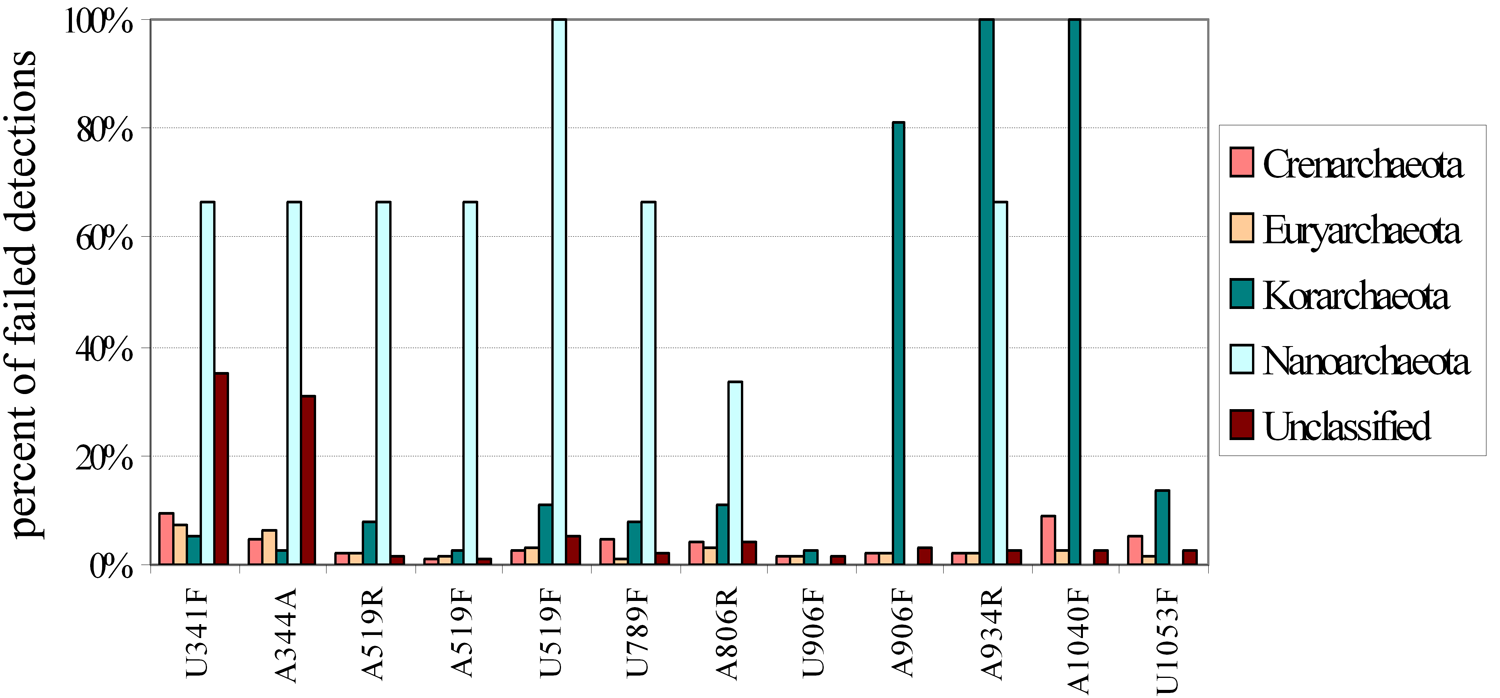

Supplement: Figure S1 — Phyla specification of known primers for archaea. Legend: The known primers were used to find their targets in a RDP dataset consisting of 1544 Crenarchaeota, 4153 Euryarchaeota, 37 Korarchaeota, 3 Nanoarchaeota and 878 unclassified species. The sequences without a target were classified into different phyla. The sequences and sources of the known primers are referred to Table 3. (3.16 MB TIF) [file pone.0007401.s001.tif]

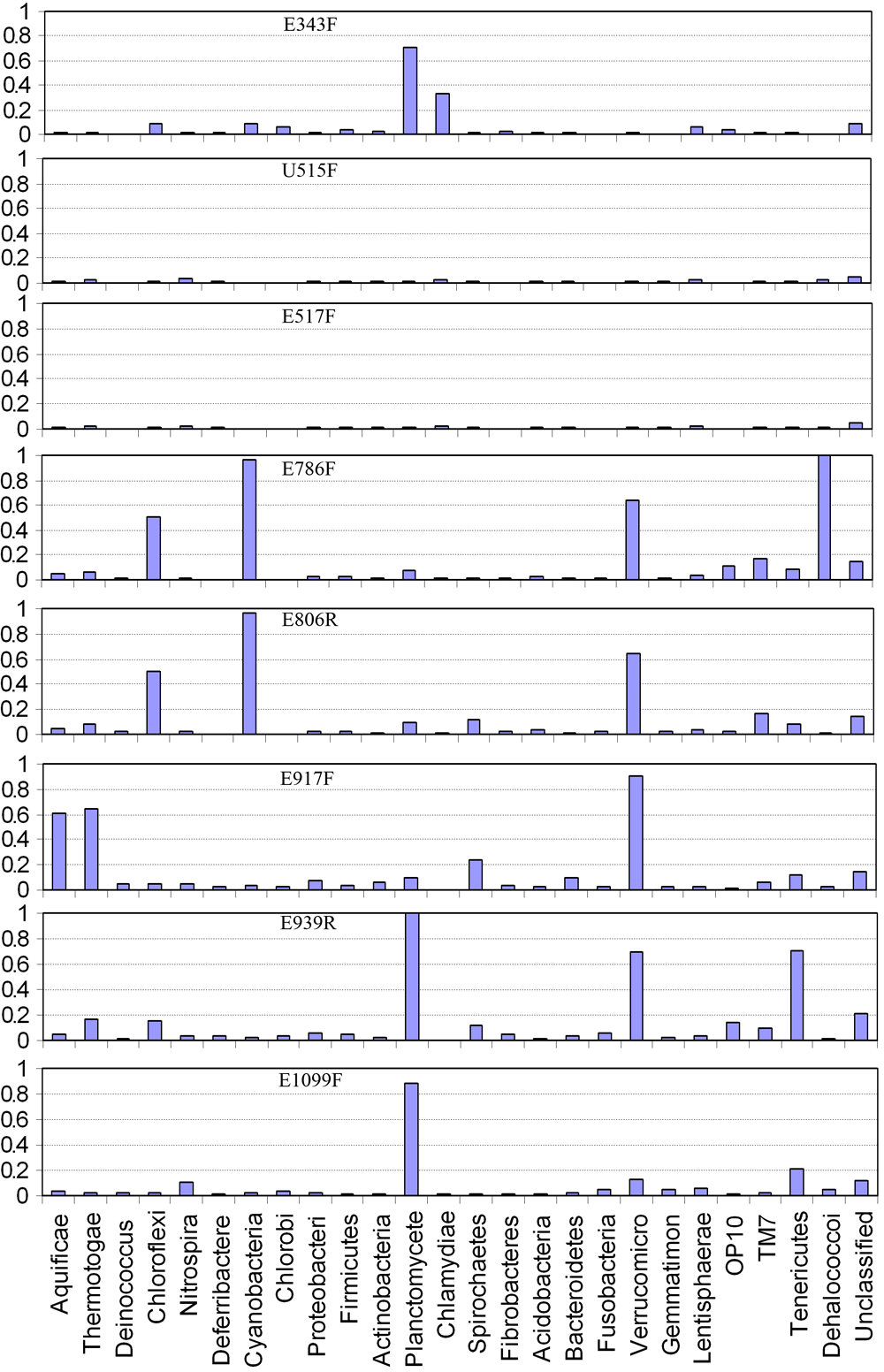

Supplement: Figure S2 — Phyla specification of known primers for eubacteria. Legend: The sequences and sources of the known primers are shown in Table 3. The details of the RDP dataset in which the primers were used to find their targets are referred to Figure 3. (4.68 MB TIF) [file pone.0007401.s002.tif]
